# Treatment of Unusually Large Acrochordon by Shave Excision and Electrodesiccation

**DOI:** 10.4103/0974-2077.41153

**Published:** 2008-01

**Authors:** Sudesh T Choudhary

**Affiliations:** *Consultant Dermatologist, A-1, Dhanjibhai Complex, Usmanpura, Ahmedabad 13, Gujarat, India*

**Keywords:** Acrochordon, electrodessication, giant

## Abstract

Acrochordons are usually small in size and are easily treated with electrofulguration under local anaesthesia. Here is a report of a giant acrochordon which posed problems in management. A technique to manage it under local anaesthesia in an office setting is described.

## INTRODUCTION

Acrochordon is an extremely common, soft, skin colored, round or oval, pendunculated papilloma.[1] It is usually constricted at the base and varies in size from 1 mm to 10 cm. Lesions tend to increase in size over a time. Acrochordon can be easily snipped or destroyed with electrocautery.[2] Here is a report of a case of giant acrochordon, weighing 2.5 kg which posed problems in management. The patient had been advised surgery under general anaesthesia which the patient had refused and was finally managed successfully in an office setting.

## CASE REPORT

A 40-year-old female presented with the history of solitary, painless and bag-like growth over the right thigh. Starting as a small tag, growth had increased to the present size in the span of 25 years. Patient had seen several doctors who had advised excision under general anaesthesia. She had refused surgery because of fear of invasive procedure.

**Figure 1:**
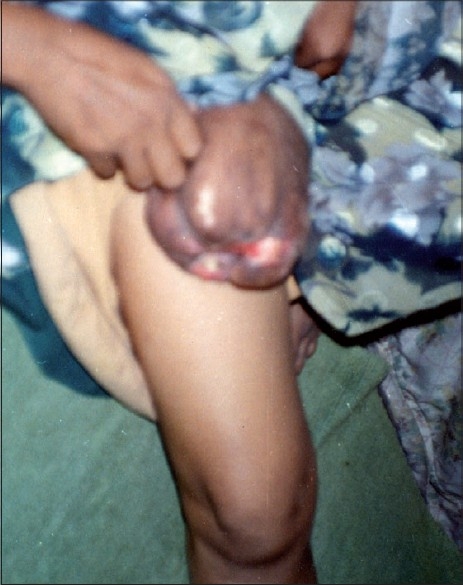
Unusually large acrochordon on right thigh

On examination, patient had a soft, non-tender, pedunculated growth measuring 6 × 7 × 8 inches over upper part of right thigh [Figure 1]. She also had several smaller acrochordons over neck and axilla. All the routine investigations were within normal limits.

Informed consent was obtained for excision under local anaesthesia. The lesion and the adjoining areas were cleaned using surgical spirit and povidone-iodine solution. Lesion was anaesthetized by infiltrating the base of pedicle by 2% xylocaine with adrenaline (1:2,00,000). Base of the pedicle was crushed and clamped by two large-sized artery forceps, pedicle was shaved parallel to the skin surface, with curved scissors.[2] Bleeding was controlled by electrodesiccation and pressure dressing.

**Figure 2:**
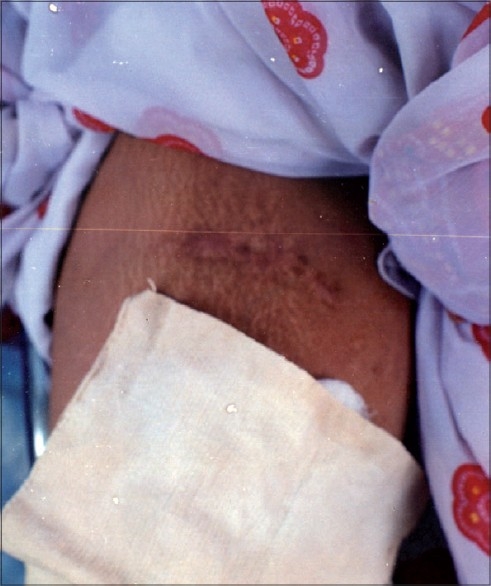
Follow-up after 18 months reveals a minor scar at the site of excision of the lesion depicted in Figure 1

Histopathology of the specimen revealed hyperkeratosis, acanthosis, loosely arranged collagen fibres. No malignant cells were seen. Follow-up after 1½ years showed a minor scar which was quite acceptable to the patient [Figure 2].

## DISCUSSION

Many patients are afraid of hospitalization and surgery because of fear of bleeding and possible complications. In the case reported herein, a giant acrochordon persisting for 25 years was completely removed, with no recurrence on follow-up for 1½ years. The minor scar left was quite acceptable to the patient. The case is reported because of rarity of giant size acrochordon (weight 2.5 kg) and very good cosmetically acceptable result by simple outpatient procedure.

## References

[1] Wolff K, Johnson RA, Suurmond D (2005). Color Atlas and Synopsis of Clinical Dermatology.

[2] Savant SS, Savant SS, Shah RA, Gore D (2005). Minor gems in dermatosurgery. Textbook and Atlas of Dermatosurgery and Cosmetology.

